# RNA motif discovery: a computational overview

**DOI:** 10.1186/s13062-015-0090-5

**Published:** 2015-10-09

**Authors:** Avinash Achar, Pål Sætrom

**Affiliations:** Department of Computer and Information Science, Norwegian University of Science and Technology, Trondheim, Norway; Department of Cancer Research and Molecular Medicine, Norwegian University of Science and Technology, Trondheim, Norway

**Keywords:** RNA, Secondary structure, Motif discovery

## Abstract

**Abstract:**

Genomic studies have greatly expanded our knowledge of structural non-coding RNAs (ncRNAs). These RNAs fold into characteristic secondary structures and perform specific-structure dependent biological functions. Hence RNA secondary structure prediction is one of the most well studied problems in computational RNA biology. Comparative sequence analysis is one of the more reliable RNA structure prediction approaches as it exploits information of multiple related sequences to infer the consensus secondary structure. This class of methods essentially learns a global secondary structure from the input sequences. In this paper, we consider the more general problem of unearthing common local secondary structure based patterns from a set of related sequences. The input sequences for example could correspond to 3^′^ or 5^′^ untranslated regions of a set of orthologous genes and the unearthed local patterns could correspond to regulatory motifs found in these regions. These sequences could also correspond to in vitro selected RNA, genomic segments housing ncRNA genes from the same family and so on. Here, we give a detailed review of the various computational techniques proposed in literature attempting to solve this general motif discovery problem. We also give empirical comparisons of some of the current state of the art methods and point out future directions of research.

**Reviewers:**

This article was reviewed by Dr. Erez Levanon, Dr. Sebastian Maurer-Stroh and Dr. Weixiong Zhang.

## Background

Classically, RNA molecules are known to be messengers from the genome for protein synthesis. Such RNA have been referred to as mRNA in short. In the last decade or so, a number of non-coding RNA (which do not code for proteins) have been discovered and the exploration for new non-coding RNA is still on [1, 2]. After the earliest and well-known ncRNAs (namely the transfer RNAs or tRNAs and ribosomal RNAs or rRNAs), a wide spectrum of ncRNAs have been discovered. These range from the short ones like microRNAs, small nucleolar RNAs and short interfering RNAs, to very long ones [3]. This development has parallely propelled the computational research in RNA bioinformatics. Computational methods have been used for a variety of tasks like secondary and tertiary structure prediction, homology search, RNA/RNA interactions, folding process dynamics, annotating ncRNA genes, microRNA identification and target prediction and so on. For a nice overview on various computational techniques in ncRNA research, refer to [4].

RNA secondary structure prediction is an old and well studied problem in RNA bioinformatics. The initial methods predicted secondary structure from single sequences and were based on approaches like thermodynamic folding [5, 6] and probabilistic modelling [7]. However these methods suffer from prediction inaccuracies and the issue can be partly resolved by comparative sequence analysis. Here, given a set of related sequences (of similar function for instance), one infers the consensus secondary structure. The idea is that these related sequences should also possess identical or very similar secondary structure. In general, RNA molecules sharing the same function typically share a combination of sequence and secondary structure similarity. There have been a number of reviews on RNA secondary structure prediction [8, 9].

The comparative sequence analysis approach for structure prediction essentially looks for a global secondary structure in a set of homologous sequences. In this paper, we focus on a more general problem of discovering secondary structure based *local* patterns from a set of related RNA sequences. We review the various computational approaches which try to solve this general problem. The task is of extracting one or more recurring patterns or motifs that are shared by a majority of the input sequences. The set of input sequences for example could correspond to the 5^′^ or 3^′^ untranslated regions (UTR) of a set of orthologous, co-expressed or co-ordinately regulated genes. These UTRs of orthologous genes are known to house a variety of regulatory motifs like Cobalamin, Lysine, glmS, Iron Response Element and the Histone 3^′^ UTR stem loop. The first three examples are specifically referred to as riboswitches [10] as certain conditions, such as interactions with small metabolites, alter their structure and thereby directly influence the production of proteins encoded by the mRNA on which they reside. The sequences could also correspond to a set of in vitro selected RNA, like the SELEX data [11]. The sequences are basically randomly generated single-stranded RNA that specifically bind to a target ligand locally owing to the existence of a common motif among the sequences. The sequences could also be a set of genomic segments containing functional structures of ncRNAs coming from the same family. The algorithms we discuss in this paper could in general, aid in discovering local unknown motifs based on secondary structure from a set of RNA sequences.

Some of the algorithms we discuss here have been used for genomic screens of ncRNA genes. There have been few reviews which survey the computational techniques addressing this problem [12–14]. Among these methods, there is a class of techniques [15–17] which takes as input a sequence-based alignment of genomes and then runs a sliding window on the alignment. In each window, every method essentially tries to learn a consensus structure in its own way. Some of these methods in principle, can be placed in the scope of this paper. However, they are sensitive to the input sequence alignments and have been extensively touched upon in the above reviews. Due to these reasons, we do not touch upon these methods in the current review. Also, our problem is a *de novo* or *ab initio* discovery of local motifs, different from the homology search problem [18]. The homology search involves looking for newer instances of a known RNA family (learnt through some known examples).

In this paper, we start off by reviewing the notion of secondary structures in RNAs and discuss the various graph-based representations of secondary structures in Section ‘RNA secondary structure’. In the next section (Section ‘Computational methods’), we describe the various existing algorithms for RNA Motif Discovery, indicating their similarities and differences from a computational perspective. In Section ‘Performance comparison’, we run some of these algorithms on data sets from the Rfam database and do a comparative experimental study of their performances. We finally conclude in Section ‘Discussions and conclusions’ summarizing the contributions of the paper.

## RNA secondary structure

RNA molecules are polymers consisting of the four nucleotides Adenine (A), Guanine (G), Cytosine (C) and Uracil (U) on a sugar-phosphate backbone. The RNA linear chain of nucleotides in general folds into a three-dimensional structure, both for the purpose of molecular stability and to perform specific biological functions.

However, the RNA molecule can be viewed in terms of what is called a “Secondary structure”. The single stranded linear RNA molecule first folds onto itself and forms double-stranded regions by additional hydrogen bonds. These double stranded regions are complementary regions as per mainly the Watson-Crick base pairings (A-U and G-C) and possible G-U pairing. These complementary base pairings lend energic stability to the molecule. RNA Secondary structure is basically a 2-D representation of this self-folding.

**Fig. 1:**
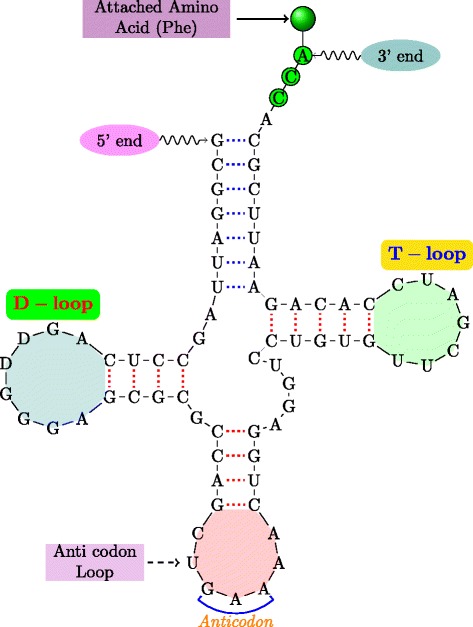
tRNA secondary structure. The tRNA is a yeast phenylalanine tRNA; annotations indicate tRNA structural elements

**Fig. 2:**
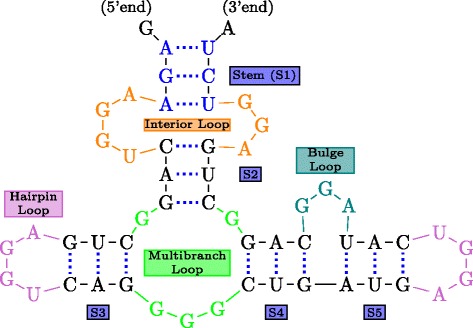
Basic Structural Motifs in RNA secondary structures. The RNA consists of five stems (S1-S5) connected by loops (color coded according to loop type)

Figure 1 shows the secondary structure of a yeast tRNA. This particular tRNA is involved in the transfer of the amino-acid phenyl-alanine to the translation site by attaching it to its 3^′^-terminal end. Figure 2 illustrates a hypothetical example of an RNA secondary structure illustrating the various basic motifs that arise in the context of RNA secondary structures. The double stranded regions formed by the stacking of two or more (maximally) consecutive base-pairs are referred to as *stems*. The single stranded regions of the secondary structure form a variety of patterns or motifs, all of which are some kind of *loops*. The *hairpin* loop is a single-stranded region that a stem ends into. A *bulge loop* can be viewed as a single stranded region which interrupts a stem on one side. An *interior loop* on the other hand interrupts a stem on either side. A single stranded region into which more than two stems meet is called *multijunction loop*. Single stranded regions in the secondary structure are in general important because they could serve as potential sites for protein/RNA binding and also be involved in additional bonding in the final tertiary structure.

**Fig. 3:**
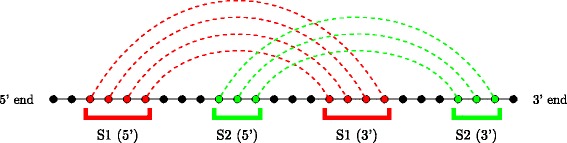
Pseudoknot definition. Circles indicate nucleotides in an RNA molecule; dotted lines show intra-molecule base-pairings. Pseudoknots involve two stems (red and green) that are interlaced such that the 5^′^ end of one stem lies between the 5^′^ and 3^′^ ends of the other stem

**Fig. 4:**
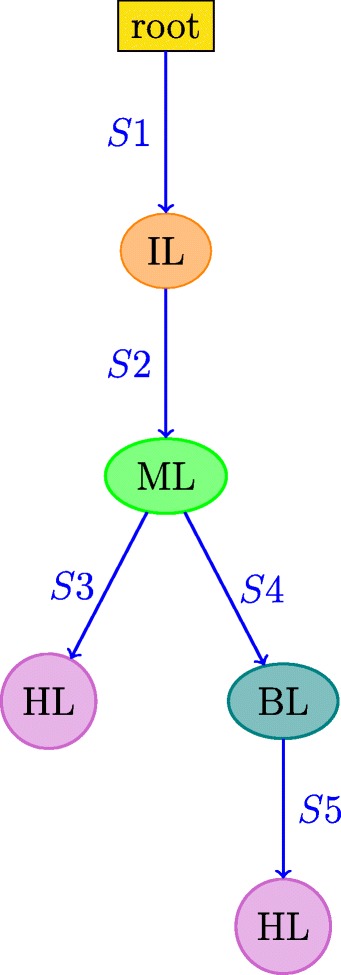
Ordered tree representation of the structure in Fig. 2. Circles and ovals (nodes) correspond to loops; arrows correspond to stems. Node colors and arrow labels correspond to the color codes and stem labels in Fig. 2

**Fig. 5:**
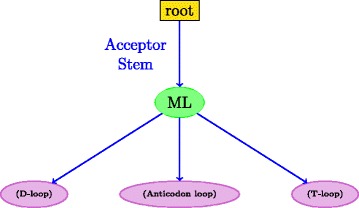
Ordered tree representation of the structure in Fig. 1. Node colors correspond to the loop types in Fig. 2; node labels correspond to loop annotations in Fig. 1

An RNA secondary structure is said to contain a *pseudoknot* if there exist two stems related as shown in Fig. 3. In a pseudoknot, the 5^′^ segment of a stem *S*_2_ is between the 5^′^ and 3^′^ segments of *S*_1_, whereas its 3^′^ segment is not. Essentially, pseudoknot indicates crossing-over of the base-pair connection lines of two different stems, such that the RNA structure can no longer be represented as a planar (2-D) graph without edge crossings. An RNA structure can be differentiated based on the presence and absence of pseudoknots. In general, handling RNA structures with pseudoknots is computationally much harder than handling RNA 2-D structures without pseudoknots as in Fig. 2. RNA secondary structures without pseudoknots have interesting tree-based representations. An ordered tree representation of an RNA secondary structure was introduced in [19]. This representation has the distinct advantage of nicely capturing the various basic secondary structure motifs discussed in Fig. 2 by denoting them as nodes and edges in the tree representation. The edges in the tree exactly capture the various stems in the secondary structure. The nodes capture the various single stranded regions like hairpin loops, bulges, interior loops and junctions. Figure 4 shows the tree representation of the secondary structure of Fig. 2. Notice that the children of any given node are ordered based on the order of their occurrence in the 5^′^−3^′^ direction of the linear sugar-phosphate backbone. Figure 5 gives another example of the tree representation. The tree represents the tRNA secondary structure shown in Fig. 1.

**Fig. 6:**
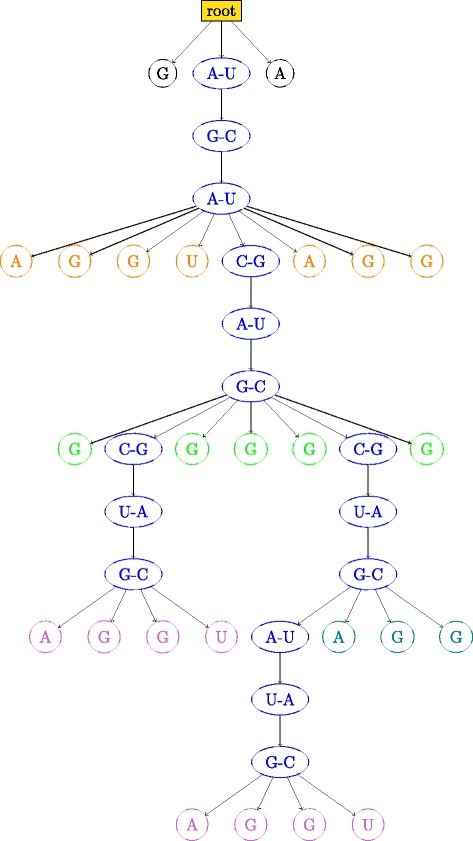
Tree representation of the structure in Fig. 2 at base and base-pair level. Node colors correspond to the color codes in Fig. 2

The above tree representation is a coarse representation of the secondary structure. The information about the size of the various basic motifs and so on are lost in this representation. One could represent secondary structures without pseudoknots as trees, without losing any information of the secondary structure [20]. In this representation, each internal node corresponds to a base-pair and each leaf node to an unpaired base. The root does not correspond to anything but is introduced to prevent the formation of a forest. The nesting created by the base pair bonds is what determines the parent sibling relationships. Figure 6 gives this tree representation for the hypothetical RNA secondary structure of Fig. 2.

**Fig. 7:**
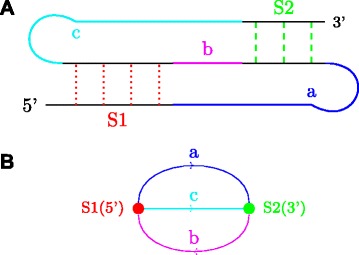
Two-Stem Pseudoknot (**a**) and its Dual Graph Representation (**b**). Stems (S1 and S2) are color coded and represented by dotted lines in (**a**) and nodes in (**b**). Single stranded regions (a, b, and c) are color coded in (**a**) and represented by arrows in (**b**)

To represent structures with pseudoknots as well, Gan et al. [21] introduce what is called the dual graph representation of an RNA secondary structure. It can be viewed as a dual of the coarse tree representation discussed before, which associates nodes with loops and edges with stems. In the dual graph representation, the stems in the secondary structure are represented as nodes and the edges in the graph come from the single stranded regions. Figure 7a is another version of the two stem pseudoknot of Fig. 3, where the stems and the various single strands are distinctly indicated. Figure 7b gives the dual graph of the two-stem (simplest) pseudoknot. Note that the dual graph representation works for any general secondary structure.

Gan et al. [21] utilize the dual graph representation to understand all the mathematically feasible RNA secondary structures possible in comparison with the already known existing secondary structures. Within the dual graph framework, Gan *et al.* [21] differentiate three types of graphs based on edge connectivity properties called trees, pseudo-knots and bridges.

Gan et al. [21] also make a thorough enumeration of the above three structure spaces and exactly indicate the structures that have been reported experimentally or in existing literature in all the three cases. Among the structures that have not yet been reported, they try to provide hints to real RNA secondary structures that could exist in nature but have not yet been discovered.

As already mentioned in the introduction, obtaining the minimum free energy (MFE) structure using efficient dynamic programming (DP) algorithms has been the earliest approach towards secondary structure prediction. The energy model adopted is additive and hence the free energy of a structure is assumed to be a sum of energies of its constituent loops [22]. Its associated thermodynamic parameters were first estimated in [23] and the estimates continue to be refined [24].

A different take on RNA secondary structures is the ensemble based approach introduced in [25]. As the word ensemble indicates, this view point considers a probability distribution over the entire space of feasible secondary structures on the given sequence. The distribution is essentially motivated from the Boltzmann distribution of statistical physics. The probability of a particular secondary structure with free energy *E* is directly proportional to *e*^−*E*/*RT*^, where *R* is the gas constant and *T* is the temperature in kelvin. The normalizing constant which makes this a distribution is referred to as the partition function. The partition function calculation is amenable to an efficient DP-based recursion.

**Fig. 8:**
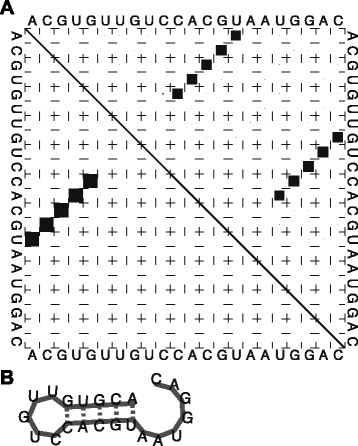
Dot plot illustration. **a** Dot plot. **b** Corresponding MFE structure (from lower left triangle in (**a**))

A very interesting feature about the Boltzmann distribution is that one can also efficiently calculate the probability of base pairing between any two positions in the sequence. The pair probability matrix between every pair of nucleotides can be nicely visualized as a 2-D plot called the dot plot [26]. At each position (*i*,*j*) in the plot there is a square whose area is proportional to the probability of the base-pair (*i*,*j*). Figure 8 gives an example of a dot plot as obtained by the RNAfold program of the ViennaRNA package [27]. The upper triangular region of the figure displays the ensemble base-pair probabilities with proportionate squares. The lower triangular region shows all the base pairs belonging to the MFE structure. As is evident, two strong helices (which cannot occur together) are clearly visible in the dot plot, while the MFE structure uses only one of the helices. The structure returned by the MFE approach is not very accurate in general. This is mainly due to factors like errors in the thermodynamic parameters and lack of knowledge of some thermodynamic rules. The dot plot clearly brings out all the highly probable stems that are feasible on folding of the sequence. Hence it can aid in visualizing various alternative structures to the MFE and exploring the correct structure among the sub-optimal ones.

The base-pair probability magnitudes have been demonstrated to be a measure of confidence for the base-pairs as predicted by the MFE structure [24]. Also, the base-pair probabilities have been found to be less sensitive to the uncertainities in the energy parameters than the MFE structure [28]. Ensemble characteristics like mean and variance of the free energy under the Boltzmann distribution have been found to be useful in distinguishing biological sequences from random sequences [29]. Given these various factors, the ensemble notion of secondary structures is very useful in general and is utilized by some of the algorithms we discuss next.

## Computational methods

The methods for RNA motif discovery can be loosely classified into four categories: (i) Stochastic (ii) Stem-based (iii) Alignment-based, and (iv) Miscellaneous. We discuss each of these classes of methods in detail in this section. We start off with the description of the stochastic methods. We wish to point out that this categorization is neither hard nor the only way to view the entire landscape of computational methods.

### Stochastic algorithms

The stochastic algorithms are essentially methods which pose motif discovery as a probabilistic learning problem. We describe three discovery methods under the category of stochastic algorithms. We start off this subsection with a brief review of the well-known DNA/Protein motif discovery tool, the MEME (multiple EM [expectation maximization] for motif elicitation) algorithm [30]. This is because two of the stochastic learning based methods for RNA motif finding are extensions of MEME. After explaining MEME, we discuss the first discovery method MEMERIS, which essentially looks for linear motifs in single stranded regions. Before moving to the next discovery method, we briefly review covariance models (CMs; a very popular tool to model secondary structure based motifs) and COVE, a method for learning a global CM from a set of unaligned sequences. Using MEME and COVE, we explain our next method, CMfinder, a state of the art motif discovery method. We end this subsection with the last method namely, RNAPromo.

#### MEME algorithm

**Fig. 9:**
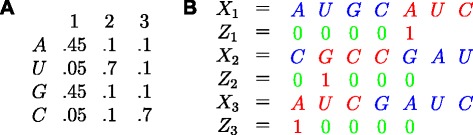
MEME illustration. **a** Position weight matrix. **b** Example sequences with motif occurrences (in red)

The MEME algorithm simultaneously learns both the motif and its location from a given set of sequences. The motif is modeled as a position weight matrix (PWM), where at each position of the motif there is a probability associated with each residue or nucleotide. For instance, a motif, which is a deterministic string with some allowed rare replacements can be easily captured by this model by choosing high probabilities to the residues in the string and assigning lower probabilities to the other residues at various positions. In the DNA context, a motif could correspond to a transcription factor binding sites pattern and the sequences could be promoter regions from co-expressed genes, promoter regions from orthologous genes from different species, chromatin immunoprecipitation (ChIP) sequence data and so on. The algorithm assumes the sequences to be generated by a probabilistic model and poses the motif discovery problem as a maximum likelihood estimation problem with hidden variables. The hidden variables are modeled as binary random variables which indicate the start of the motif location. Figure 9a illustrates a 3-length motif. Figure 9b gives an example with three sequences and an occurrence of the motif (from Fig. 9a) in each of them. The *i*^*th*^ sequence is denoted as *X*_*i*_ and its corresponding hidden binary sequence (encoding the motif location) by *Z*_*i*_. The set of positions where there are no motifs (also referred to as background), is modeled as an independent and identically distributed (i.i.d) random process.

To start off, a simple model which assumes exactly one motif occurrence per sequence (referred in short as OOPS) is considered. MEME employs an EM algorithm for learning, which in general is a popular approach when the model has hidden or unobserved variables. The EM algorithm is an iterative algorithm involving two steps at each iteration. The first step called the *E* step involves calculating the expected value of the hidden variables given the current parameter values (PWM values in this case). The second step in general involves calculating the new set of parameters by maximizing a suitable function given the expected value of the hidden variables. It turns out that there exists a closed form solution to this maximum in terms of the expected value of the hidden variables. On convergence, the hidden variable whose expectation is highest in a given sequence gives us an estimate of the motif position. Two variants of this model are also considered. The first variant considers the number of motif occurrences to be zero or one (referred in short as ZOOPS model), whereas the second variant considers zero to a finite number, *n*, of occurrences.

#### MEMERIS algorithm

In the MEME algorithm, specifically for the OOPS model, there is an important underlying assumption with regard to the data likelihood calculation. This assumption is that the unconditional (or prior) probability of a motif occurrence in any position of a given sequence is the same. In other words, the probability of any of the hidden variables being one in any given sequence is the same. Hence, mathematically, the likelihood for the OOPS model is

$$\begin{array}{@{}rcl@{}} P(D/p) &=& \prod_{i=1}^{n}P(X_{i}/p)  \\ &=& \prod_{i} \sum_{j} P(X_{i}/Z_{ij}=1)P(Z_{ij}=1)  \\ &=&(L-w+1)^{-n}\prod_{i}\sum_{j} P(X_{i}/Z_{ij}=1)  \end{array} $$

In the above equations, *D* is the entire set of sequences, *L* is the length of each sequence (assumed same here for convenience of illustration), *n* is the number of sequences, *w* is the length of the motif and *p* is the PWM. Note that *P*(*Z*_*ij*_=1)=1/(*L*−*w*+1)∀*j*. For the ZOOPS model, this translates to saying that if a motif occurs in a given sequence it occurs with uniform probability in any of the positions.

The first of the RNA motif finding algorithms we discuss here is referred to as MEMERIS (MEME in RNAs including secondary structures), proposed in [31]. As the name suggests, it basically uses an MEME like algorithm by utilizing non-uniform prior probabilities (instead of uniform) on the probable position of the motif occurrences. The algorithm tries to discover sequence specific motifs which could correspond to binding sites for proteins in mRNA. Such sites are often located in the 3^′^ or 5^′^ UTR of mRNAs and more specifically in the single-stranded regions. The problem posed like this boils down to sequence motif discovery localized to single stranded regions of the RNA. Towards this, MEMERIS first calculates the probabilities in the Boltzmann ensemble of every sub-string (of some fixed length *w*) being single stranded. For this calculation it utilizes the partition function version of RNAfold [27]. These probabilities for a given sequence are then suitably normalized to obtain a distribution over the possible motif positions. This is in line with the intuition that one would want to weight the probability of a motif occurring at a given position proportional to the probability of the sub-string beginning at that position being single stranded, as one is looking for motifs in single stranded regions here. Hiller et al. [31] demonstrate how the algorithm actually avoids identifying patterns that exist in double-stranded regions of RNA molecules both in real and synthetic data.

#### Review of covariance models and COVE algorithm

**Fig. 10:**
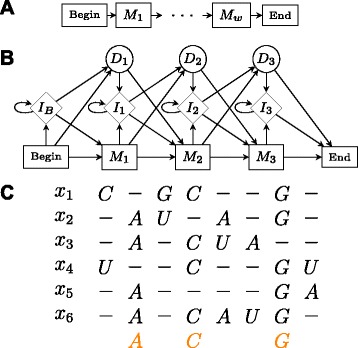
Profile HMMs. **a** A trivial HMM corresponding to a PWM of length *w*. **b** A profile HMM with insert (*I*_*i*_), delete (*D*_*i*_), and match (*M*_*i*_) states. **c** Multiple alignment that could correspond to the HMM in (**b**)

CMfinder [32], the next algorithm we intend to discuss, extends MEME in a different and more involved way as compared to MEMERIS. CMfinder essentially generalizes the motif structure considered in MEME. In MEME, the PWM motif is basically a string of characters which also takes into account a few replacements. A PWM motif of size *w* could be viewed as a hidden Markov model where the underlying Markov chain has *w* states $M_{1}, M_{2}\dots M_{w}$ as shown in Fig. 10a. There is a simple linear state transition structure from *M*_1_ to *M*_*W*_ with *M*_*i*_ transiting to *M*_*i*+1_ with probability 1. The emission probabilities at a state *M*_*i*_ would correspond to the *i*^*th*^ column of the PWM. Profile hidden Markov models (HMMs) generalize this simple model to incorporate insertions and deletions by adding insert and delete states suitably to this stochastic model. In other words, Profile HMMs are stochastic models which nicely capture salient features of a set of multiply aligned sequences. Figure 10b gives an example of a profile HMM with three match states.

**Fig. 11:**
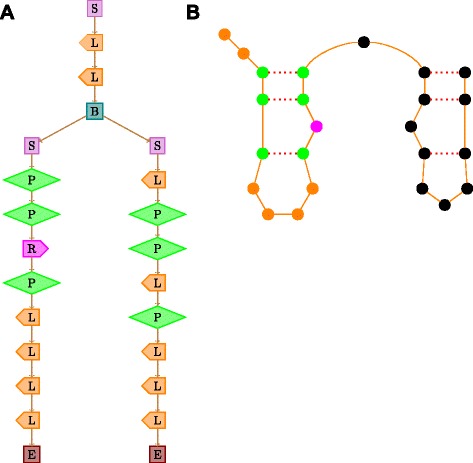
Covariance Models (CMs) without insertion and deletion states. **a** CM tree and its (**b**) secondary structure. Colored circles in (**b**) correspond to the states from the root start state (S) to the left leaf end state (E) in (**a**)

Figure 10c is an example multiple alignment from which the profile HMM given in Fig. 10b could be learnt. Covariance models generalize profile HMMs to cater to both sequence and structure similarity. These are a class of stochastic context-free grammars (SCFGs)[33] where the underlying hidden process need not be a Markov chain. Hence, unlike profile HMMs, the state transition structure among the match states *M*_*i*_ need not be linear (in fact, it is a tree structure in general) and these match states can now even emit pairs of symbols in order to capture base pairing in RNA secondary structures. Figure 11a gives an example of a CM with a tree like state transition structure of matched (or consensus) states without insert or delete states. The states marked *P* emit a pair of bases and in general can emit the 16 possible base-pairs. The states marked *L* and *R* emit a single base and are similar to the match states of the profile HMM. The *L* and *R* distinction differentiates the leftwise and rightwise non-terminals from a grammar perspective. The state marked *B* is a bifurcation state which captures the splits in secondary structures due to multi-junction loops or multiple stems. The state *S* refers to the start of the process OR starts of substructures that a bifurcation state divides into. There are no symbols emitted from states *S* and *B*. The state *E* generates *ε* with probability 1. Figure 11b gives the corresponding secondary structure that is exactly captured by the tree of Fig. 11a. The positions and the corresponding states capturing them are marked in the same colour. A CM will in general have additional insert and delete states like the ones shown in Fig. 10b and more in order to capture insertion and deletion of base-pairs also. Covariance models were introduced in [34] as a convenient stochastic model to capture the common global secondary structural features of a family of RNA sequences. The idea is that the underlying tree of match states basically captures the consensus secondary structure common to all sequences in the family. They propose COVE, an interesting iterative algorithm for learning a global CM from a set of unaligned sequences based on comparative sequence analysis.

Each iteration in the algorithm involves two steps: (i) Given the current alignment, build an optimal CM and (ii) given the current CM model, find the optimal multiple alignment between the sequences. Essentially, the algorithm is trying to learn the structure and the associated parameters simultaneously. The first step is achieved by a DP algorithm very similar to the Nussinov algorithm [5] which for a given RNA sequence computes a secondary structure (without pseudo-knots) with the maximum number of base pairs. Under the cost function used here, a position pair (*i*,*j*) has a cost of either 1 or 0 depending on whether the residues at the position *i* and *j* are Watson and Crick base pairs. The COVE tool uses a generalization of this 0−1 cost function, namely mutual information between positions *i* and *j* which can be empirically measured from the current multiple alignment. The obtained consensus structure is then matched with every aligned sequence to obtain separate parse trees. Empirical counts of various transitions and emissions are used in estimating the probability parameters of the CM. Regarding the second step, a Viterbi-like DP algorithm is applied on each sequence to obtain the most likely set of states in the CM. A multiple alignment is now obtained by the individual alignments of the sequences to the common CM.

#### CMfinder

CMfinder [32] combines MEME and COVE with some heuristics incorporated. The procedure starts off by constructing an initial alignment exploiting the sequence information in a heuristic manner. Towards this, it first selects a set of potential candidates. It achieves this by computing the minimal free energy of all sub-sequences in a given sequence using RNAfold of the ViennaRNA package [27]. The sub-sequences whose length is in the range 30 to 100, which have 1−2 stem-loops and are locally optimal (in the sense of no lower-energy states by deleting or extending 2 bases at the end) are chosen.

The goal of the algorithm is to find local patterns which occur in a sufficient majority of the sequences. In line with this, the next step tries to group together similar potential candidates from different sequences and form a set of alignments. For this, it first computes a pairwise distance matrix for all candidates. This distance matrix is calculated by comparing two sequence trees at the base/base-pair level (as in Fig. 6) by using a modified version of the tree-edit algorithm implemented in RNAdist of the ViennaRNA package. Using this distance function for every candidate it computes the closest candidate to it in every other sequence and assigns a score equal to the sum of each of these distances. A candidate with the least score is chosen as a consensus candidate. This is used as a seed to group in candidates from other sequences similar to this in an iterative fashion. Given the current candidates in the expanding group, it finds a candidate from the remaining sequences whose average distance to the sequences in the current group is minimum. If this minimum is less than a certain threshold, then it adds this candidate to the group. Otherwise, the process terminates. The thresholding allows for the possibility of the motif being absent from some sequences. The initial alignment is constructed based on a pairwise tree-alignment between each chosen candidate and the consensus candidate. One can now choose the next “consensus candidate” from the unchosen candidates and build subsequent alignments.

Given the initial alignment one can estimate a CM as explained before to start the EM iteration. The EM algorithm is applied with ZOOPS as the underlying generative model. In a given RNA sequence, the probable motif occurrence positions are assumed to be uniformly distributed only over the start positions of all the potential candidates extracted from the sequence instead of all positions as before. The *E*-step involves estimating the probability of a motif occurrence at these candidate start position (the expected value of hidden variables explained before), which is similar to MEME. The new aspect in this step is the computation of an alignment of each candidate sequence to the current model. The alignment of a candidate sequence to the current CM is achieved by a DP algorithm [34] similar to the Viterbi algorithm. The probability of a motif occurring at a given candidate position given the current CM is carried out approximately but more efficiently using the optimal alignments computed just before. The *M*-step involves updating the model and *γ*, the probability of motif occurring in a sequence. The *γ* update is very similar to MEME. The model update step is not straightforward as in MEME and utilizes the ideas of COVE to the fullest. The first step would be to infer the new consensus secondary structure from the updated alignment of all candidate sequences in the *E*-step. In the cost function formulation, CMfinder utilizes a combination of the mutual information (as in COVE) and partition function [25]. The partition function based *P*_*ij*_, which captures the probability of the *i*^*th*^ residue pairing up with the *j*^*th*^ residue in any given sequence is used to capture an informative prior on the structures. The informative priors are calculated essentially by averaged values of the partition function across the candidate sequences. Both the mutual information and informative prior calculation involves averaging across the candidate sequences. This averaging of the candidate sequences is further weighted based on the probability of these being motif instances, a quantity calculated in the *E* step.

#### RNApromo

The last stochastic algorithm we discuss here, referred to as RNApromo was proposed in [35]. This algorithm assumes that one has information about the RNA secondary structure. The motif used here is based on an SCFG similar (but slightly different) to the CMs considered by CMfinder. The local motif finding problem is posed as an SCFG learning problem. Dynamic programming based algorithms for learning SCFGs like the inside-outside algorithm [33] exist and are analogous to the forward-backward algorithms of HMMs.

RNApromo assumes that there is secondary structure information available of all the input RNA sequences. The learning involves an initialization step similar to CMfinder. It exploits the secondary structure information in coming up with short candidate structures that occur in the majority of the input RNA structures. Each of these structures are used to initialize a separate model. The model being similar to the CM discussed earlier has a tree structure of states (similar to Fig. 11) and some additional delete and insert states around each of the tree states. The short candidate structures initialize the tree part of the model. The remaining parameters which include the various emission and transition probabilities are learnt by a modified version of a DP-based algorithm proposed in [36] for learning SCGF parameters (similar to the inside-outside algorithm). Each RNA input here contains both the sequence and structure information. This makes the likelihood computation more efficient as the number of relevant productions that generate an input now is lesser than with an input containing a sequence alone. As shown in [35], this restriction also reduces the running time of the model estimation algorithm from ${\mathcal {O}}(L^{3})$ to ${\mathcal {O}}(L^{2})$, where *L* is the length of the RNA sequence.

### Stem-based algorithms

**Fig. 12:**
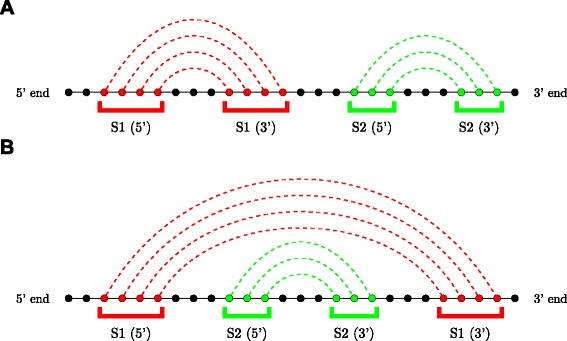
Stem relations. **a** Parallel and (**b**) nested stems. Circles indicate nucleotides; dotted lines show intra-molecule base-pairings

**Fig. 13:**
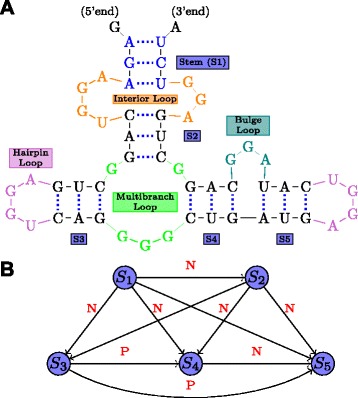
Secondary structure (**a**) and its associated stem graph (**b**). Nodes in (**b**) represent the corresponding labelled stems in (**a**). Edge labels in (**b**) indicate whether the stems are parallel (P) or nested (N)

The two methods we discuss in this category use the most likely stems of the input RNA sequences for motif discovery. The methods unearth stable stems using methods like dot matrix analysis or the partition function approach [25]. The extracted stems turn out as the common starting step of these methods. Note that the extracted stems could overlap. The RNA motifs (or patterns) here are viewed as a graph of stems, where edges are directed and can have three kinds of labels. The three labels come from the three ways in which any two stems could be related: (i) parallel – the 5^′^ and the 3^′^ strands of one of the stems is to the left of the 5^′^ strand of the other in the sequence (Fig. 12a), (ii) nested – the 5^′^ and the 3^′^ strands of one of the stems lies within the strand connecting the 5^′^ and 3^′^ strand of the other stem (Fig. 12b), and (iii) pseudo-knotted (see Fig. 3, Section ‘RNA secondary structure’). The pattern graph is a completely connected directed graph. Figure 13b gives an example. Any two stems in the pattern must be related in one of the above 3 ways. The direction of an edge would be from the stem whose 5^′^ strand is more upstream in the sequence. The common goal of both the methods is to extract stem graphs which occur frequently enough in the input RNA sequences. Each node in the stem graph would actually represent a group of similar stems (each stem in the group coming from a different sequence). This similarity between stems is captured based on features like stem length, sequence, stem stability, loop sequence closed by the stem and so on. Another advantage about these algorithms is that they can unearth motifs with pseudo-knots. We now explain the two algorithms in detail.

#### comRNA algorithm

The first method we discuss is comRNA, proposed in [37]. As a first step, comRNA extracts groups of similar stems (each from a different sequence). Towards this, it extracts pairs of similar stems based on features like helix length and so on discussed above. It forms a graph with all stems as nodes and two stems connected if they were found similar. Maximal cliques from this graph are extracted which would now form the groups of similar stems that the first step extracts. A group of similar stems which is conserved across multiple sequences is referred to as a stem block. As explained earlier, each stem block here corresponds to possible occurrences of an individual node of the stem graph pattern discussed above. Finding an assembly of these stem blocks from various sequences is the final objective. The next step would be to build a directed graph where each node of the graph corresponds to a stem block. An edge is drawn from a stem block *b*_1_ to a stem block *b*_2_ if stems from *b*_1_ are before that of *b*_2_ and form one of the three previously described consistent patterns with the stems of *b*_2_ in a sufficient number of sequences. The last step is to extract maximal paths in this directed graph in a recursive depth-first fashion. Each such maximal path would correspond to a stem graph patterns. While recursively growing the paths of stem blocks, one must also make sure that all currently included stem blocks must occur together in at least a user-defined number of sequences.

#### RNAmine algorithm

**Fig. 14:**
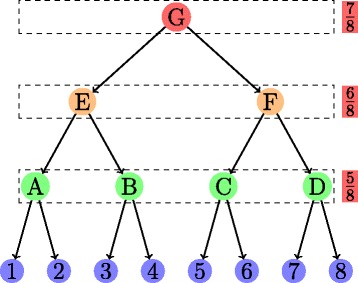
RNAmine label tree. Dashed boxes indicate layers; red boxes represent layer costs

As opposed to comRNA which uses several ad-hoc steps, RNAmine proposed in [38] uses a more systematic approach using graph mining ideas to extract stem patterns. The subarea of graph mining [39] is pretty mature within the area of frequent pattern mining. In RNAmine, each input sequence [38] is viewed as a directed graph of stems (extracted from the sequence). The edges in this graph are based on the three cases discussed above and have a label associated with them depending on the type of association. Note that since the stems obtained in a sequence can overlap in general, the stem graph of an input sequence is not a connected graph in general. The algorithm considers a slightly different distance measure between stems compared to comRNA, which again is some weighted combination of different features. Based on this distance measure, the grouping of stems is performed by clustering in a hierarchical fashion. A label is assigned to each cluster at each layer and these labels can be arranged as a tree as shown in Fig. 14. The nodes of the stem pattern here are taken from arbitrary levels of this tree. A stem pattern *P* matches a directed graph *G* if both have the same topology and edge labels, and every node label in *P* is an ancestor of the label of the corresponding node in *G* as per the label tree. The problem of extracting meaningful patterns is solved as a constrained graph mining problem. One obvious constraint is that the extracted graphs cannot be general graph patterns, but must be cliques as any two stems in our pattern must be related. The other constraint pertains to the generality of the pattern from the labels perspective. A pattern having too many labels from the top of the label tree is undesirable as such patterns do not convey much information. Hence, to direct the search to use labels from the lower layers, a cost which is an increasing function of the layer height is defined as indicated at each layer in Fig. 14. The cost of a pattern is defined as an average cost of the labels of all its nodes. Given this, the third constraint is an upper bound on this cost. The interesting thing to note is that in addition to the frequency of the pattern (common in any frequent pattern mining algorithm), the new cost of the pattern could also be utilized in pruning the exponential search space during actual mining process.

### Alignment-based methods

The search for a common local pattern among a set of RNA sequences can be cast as a multiple local alignment problem, where the alignment takes into account both sequence similarity and base-pair correlation. There have been many methods based on local alignment of RNA sequences. The methods under this category can be loosely classified into two categories: (i) secondary structure independent and (ii) secondary structure dependent. The secondary structure independent methods just need the sequence information of the input sequences. Secondary structure dependent methods additionally need the secondary structure information of the input RNA sequences. We now discuss both these classes of methods.

#### Secondary structure independent methods

FOLDALIGN, proposed in [40], is the first algorithm we discuss in this category. This was also one of the earliest algorithms trying to discover secondary structure based local patterns from RNA sequences. The algorithm basically uses a 4-dimensional recursion to obtain the local alignment of a set of sequences. The Sankoff algorithm [41] is the first DP algorithm which tries to simultaneously align and predict the structures of two or more RNA sequences in a global way. For a pair of sequences of length *L*, it uses a 4-dimensional recursion with a time complexity of ${\mathcal {O}}(L^{6})$ and a space complexity of ${\mathcal {O}}(L^{4})$. The DP recursion in [40] is motivated from this. The Sankoff algorithm tries to minimize the sum of alignment distance and free energy of the involved RNA molecules. In contrast to this, Gorodkin et al. [40] maximize a score which is a combination of sequence similarity (local) and the number of base pairs. It essentially combines the two dimensional Smith-Watermann (local alignment) and the Nussinov algorithm. For efficiency purposes, they skip the branching part of Nussinov recursion to achieve a ${\mathcal {O}}(L^{4})$ time complexity. But this leads to the algorithm being restrictive in the sense that it only searches for stem-loop motifs. Given a set of *N* sequences, FOLDALIGN tries to come up with a subset of sequences which contain the most significant common motif. The idea is to be able to exclude a set of sequences which do not belong to the same structural class. Since the number of such subsets of sequences can be exponential in the number of sequences, a greedy algorithm which constructs an *r*-sequence alignment by aligning a single sequence with an (*r*−1) sequence alignment is proposed. To achieve this, the aligned columns within an existing alignment are viewed as a single entity and the score between two columns is essentially the sum of all pairwise scores. An improvement on their greedy strategy to make the discovery faster was proposed in [42]. An improvement in the discovered secondary structure by combining it with COVE (described earlier in Section ‘Review of covariance models and COVE algorithm’), is achieved in [43]. The local alignment learnt by FOLDALIGN is used as a seed alignment to be improved upon by COVE. A pairwise local alignment algorithm which can discover any branched but non-pseudoknotted structure was introduced in [44]. The scoring function in this new version of FOLDALIGN uses a context dependent (like the different loops and stems) free energy instead of just base pairs of the earlier version. An upper bound on the length of the pattern and a maximum length difference between subsequences compared leads to a very efficient algorithm.

Another approach for multiple local alignment has been to use the pair probability matrices of sequences which capture the probability of base pairing between any two nucleotides in a particular RNA sequence. These pair probabilities are computed based on a full energy model using McCaskill’s algorithm [25]. The algorithm computes the alignment and secondary structure simultaneously. The score function to be minimized captures the base-pair scores (which are now a function of pair probabilities), similarity score for matches, mismatches and gaps. The DP recursions are similar to the Sankoff algorithm [41]. LocARNA assumes a cut-off below which the pairing probabilities are ignored. The number of significant entries (above the cut-off) for a particular base remain constant as the length of the sequence increases. This lower bound results in both timewise and spacewise efficiencies (for eg: time complexity reduces from ${\mathcal {O}}(L^{6})$ to ${\mathcal {O}}(L^{4})$). A progressive multiple alignment method is now constructed based on the pairwise alignment algorithm LocARNA. Towards this, one would need a consensus base-pairing probability matrix for the combined alignment of two subalignments. This is suitably defined using roughly the geometric mean of the corresponding probabilities of the constituent subalignments.

Tabei and Asai [45] propose an approach SCARNA_LM where they model alignments using a conditional random field (CRF). The underlying model structure used for pairwise local alignments is quite intuitive. The local alignment is modelled using three states. A state *M* models the matched nucleotides of the alignment, whereas the remaining two states capture the inserts into the two sequences respectively. Then there are states which capture the flanking regions of the alignment. The CRF here models the conditional probability of the alignment given the pair of sequences to be aligned. This modelling is based on certain local features (as in any CRF) which capture secondary structure information in the form of base-pairing probabilities. They basically condense the probability of a particular base pairing to any other nucleotide into three probabilities, namely, probability of pairing to some nucleotide to its left, right, or remaining unpaired. These three probabilities are suitably used to define local feature values. The final alignment algorithm tries to find the local alignment which maximizes a suitable cost function based on a *γ*-centroid estimator. The cost function contains terms involving probability of two bases (one from each sequence) being aligned in the alignment. These probabilities are pre-calculated based on the described CRF using a forward-backward algorithm. This cost function is maximized and an alignment computed using a Smith-Watermann recursion. Note that this alignment doesn’t explicitly yield any base-pairing information in the locally aligned subsequences, even though the base-pairing probability information is utilized in arriving at the alignment. Using this pairwise alignment algorithm, SCARNA_LM computes pairwise local alignments for each pair of sequences. Next, it generates blocks of possibly alignable regions from the pairwise local alignments similar to an approach in [46]. The sequences in each block are aligned using MXSCARNA [47], a secondary structure based global alignment algorithm which yields the final multiple alignment.

#### Secondary structure dependent methods

A pairwise alignment method and its extension tackling multiple alignments is considered in [48]. The pairwise alignment algorithm takes as input two RNA sequences along with the secondary structure information. The secondary structure is essentially viewed as a sequence of what are called structure components. These can be either single bases or base pairs. So an alignment is basically viewed as between two structure component sequences. While looking for an optimal alignment, the algorithm looks for certain constrained alignments based on what they call a hierarchical constraint. The hierarchy is based on a tree of circles for each sequence. Each structure component is roughly mapped to a unique circle. The tree of circles is quite similar to the tree representation discussed earlier in Figs. 4 and 5 of Section ‘RNA secondary structure’. Each of the single-stranded motifs like loops and bulges which are nodes of the ordered tree representation correspond to a circle in the tree discussed in [48]. However there are circles which capture base pairs of the secondary structure, which were otherwise lumped as edges in the ordered tree representation of Figs. 4 and 5. Based on the tree of circles for a sequence, any two structure components are hierarchically related to each other in three possible ways: (i) ancestor/descendent (ii) common ancestor (iii) identical circle. Hence, in an alignment if (*A*_*i*_,*B*_*i*_) and (*A*_*j*_,*B*_*j*_) are matched component pairs, then the hierarchical constraint between *A*_*i*_ and *A*_*j*_ must be the same as that between *B*_*i*_ and *B*_*j*_. The DP algorithm intelligently incorporates this constraint in the recursion.

Höchsmann et al. [49] consider a local alignment based method to discover common local motifs from a pair of RNA sequences. They consider the forest representation of secondary structures at the base/base-pair levels as discussed in Fig. 6 without the root nodes. In fact, their forest representation is a slight extension over this. They consider this fine representation over the coarser one (containing stems and different kinds of loops), as defining score functions at the base/base-pair level is more natural than between the features like stems and loops. They build on the dynamic-programming based pairwise global tree alignment algorithm proposed in [50]. It is interesting to note that unlike sequences, in the case of trees, the edit distance and alignment problems are not equivalent. For an excellent review on edit distance of trees, tree alignment and related problems, refer to [51]. The idea of a local alignment between two input structures is to look for two substructures with maximal similarity. In the case of strings, typically we look for two maximally similar substrings. However, one could also look for maximally similar suffixes in strings. Höchsmann et al. [49] consider the analogue of suffixes in forests, what are called *closed subforests* as the substructures of interest. They basically pose the local alignment problem as a local closed subforest similarity problem. Extension of these tree alignment based ideas to a multiple alignment setting was considered in [52], but this unfortunately cannot handle local alignments.

The local closed subforest similarity considered in [52] restrict the aligned region to consist of a contiguous subsequence (substring) from each of the two input sequences. This is in fact a restriction and there exist biologically meaningful motifs which violate this. An example is the mRNA element SECIS (selenocysteine insertion sequence) which binds to the protein Se1B. Backofen and Will [53] consider a local alignment which captures such non-contiguous motifs. As they describe, the local patterns that they tackle should form a connected subgraph of the input sequence-structure graph. The sequence-structure graph is a graph that has edges between two adjacent elements of the sugar-phosphate backbone and also between nucleotides involved in basepairing. They propose a DP algorithm to compute (more general) optimal local alignments between a pair of RNA sequences with known structures.

In [54], an algorithm to extract a certain kind of local matching patterns all along the input pair of sequences is proposed. They specifically consider alignments without explicit capture of insertions or deletions. The patterns they define are called matchings which are essentially a pair of aligned subsequences from the two input sequences. The matching that are considered have to be additionally connected and bond-preserving. This means, the respective subsequences of a matching have to be a connected subgraph (in terms of the backbone or base-pair edges) as in the previous method and for any two nucleotides that bond in a sequence (either via the backbone OR base-pairing), the associated nucleotides (as per the matching) in the other sequence must bond accordingly. The pattern discovery task is posed as a problem of extracting all (possibly more than one) non-overlapping, maximally extended, bond-preserving matchings.

### Miscellaneous

The next method we discuss is based on a pattern mining approach. As mentioned earlier, the data mining literature has seen a vast development in the subarea of graph mining [39]. In particular, there have been algorithms which mine for graph patterns as specific as trees. If one exactly knows the secondary structures of an RNA molecule (either experimentally or computationally), then the problem of finding consensus patterns among a set of RNA sequences can be cast as a problem of mining frequent tree patterns from a set of trees (secondary structures of RNA molecules). Zaki [55] proposes an efficient algorithm for mining frequent trees and considers mining RNA motifs from tree-structured RNA secondary structures as an example application. This is a very natural application for tree mining. A pattern tree can be matched with a data tree at different levels of generality. An occurrence could mean that the pattern tree occurs as a connected sub-graph of the data tree. More generally, an occurrence could mean the pattern tree occurs as a sub-graph of the data tree in the sense that if node *b* is a child of node *a* in the pattern tree, then the node in the data tree associated with node *b* need to only be a descendant of *a*.

RNAProfile [56] is a relatively simple method with a heuristic approach. The approach in principle is very similar to the initialization step of CMfinder to build the initial alignments. It also takes as input the number of hairpins contained in the motif of interest. It is a two step process. The first step involves extracting a set of candidate regions from each input sequence (satisfying the number of hairpins constraint) by folding the regions optimally using some prediction tool. The second step tries to group together similar candidate regions from each sequence. Since there are an exponentially number of such possibilities, it uses a greedy heuristic algorithm which starts by comparing regions between two sequences. It only retains and uses pairs of regions with high similarity for further consideration with regions of the third sequence and so on.

#### Genetic programming-based methods

The literature has also seen algorithms based on genetic programming, a methodology motivated by biological evolution. We discuss two methods GPRM [57, 58] and GeRNAMo [59] under this approach. The general style of genetic programming algorithms is as follows. They start off with a population of individuals or candidate solutions. The algorithms involve a very careful design of what is called a fitness function which assigns a score to each individual. Genetic programming essentially tries to stochastically optimize this fitness function over the space of all solutions. The strategy at each step involves using the current generation of individuals to suitably generate the next generation. Inspired by natural selection, individuals with a high fitness are more likely to be chosen from a given generation than are individuals with a low fitness. Being evolutionarily motivated, these selected individuals are subjected to genetic operations like cross over and mutation.

Each individual or candidate here corresponds to a putative motif. The motif structure is essentially same in both GeRNAMo and GPRM; specifically, an RNA motif is represented as a sequence of segments. Each segment is either single stranded or a Watson-Crick complementary segment. Each segment also bears information about the upper and lower bound on its length. For instance, *h*5(3:4)*ss*(4:6)*h*3(3:4) represents a stem-loop motif with a stem length of 3 to 4 base-pairs and a loop of size between 4 and 6. If one is looking at only non-pseudo knotted structures as in GeRNAMo, then this representation is unambiguous. GPRM considers structures with pseudoknots as well and hence needs an added index information on every complementary segment about its pairing segment. In GeRNAMo, the above described motif is represented as a tree (different from everything we have discussed so far) basically for the purpose of increased sensitivity and diversity on application of the genetic operators. For instance, the minimum and maximum length of each segment is not represented using a single number, but distributed into a tree containing the numerals ^′^0^′^, ^′^1^′^, ^′^2^′^ and ^′^3^′^. The sum of all these numerals in this subtree would capture the bound of the segment.

In the given set of input sequences, GPRM additionally expects information about the presence or absence of a motif. This labelling information is crucial in defining its fitness function. The idea is that motifs which occur mainly in the positive sequences and hardly in the negative ones are assigned high fitness. Fitness in GeRNAMo is chosen such that motifs which occur in a majority of the sequences and has the most segments have a higher fitness score. The mutation operator for GPRM first chooses a random segment from the motif. If the segment is single stranded, then its length range is randomly changed. If the segment is a complementary segment, then not only its length range but also the position of its pairing segment is randomly changed. This can be done as GPRM admits pseudoknots. In GeRNAMo, the mutation is carried out by randomly choosing a node from the tree representation of the motif described above, deleting the subtree rooted at it and growing a new one. In GPRM, the cross over operation as the name suggests involves a crossover of two randomly chosen segments between two relatively fit individuals. For a meaningful output, the paired complementary segments are viewed as a single unit. For cross over in GeRNAMo, the operation is similar. But given the tree representation, one needs to take additional care to exchange compatible parts only. While checking for occurrences of a pattern, GeRNAMo adopts the following strategy. From each sequence, it first chooses subsequences of lengths varying between the user-given minimum and maximum lengths. On each of these segments, RNAsubopt [60] is applied to obtain a set of suboptimal secondary structures. A motif’s occurrence is checked by comparing its segment based representation with each subsequence’s suboptimal secondary structures. On the other hand in GPRM, stems in each sequence are first unearthed. The occurrence of a motif is checked by essentially comparing the motif’s complementary segments with the stems in the sequence.

## Performance comparison

In order to compare some of the main algorithms, we consider four families from Rfam [61] (release 11.0). The families were chosen with a varied motif length (about 70 to 175) and a variation in depth and branching of the motif tree (tree representation with nodes being basic secondary structure motifs as in Fig. 4 discussed earlier). All the chosen (four) families are regulatory motifs housed either in the 3^′^ or 5^′^ UTR regions of mRNA. We work with seed sequences in each family and use a maximum of 20 sequences per family.

In our benchmarks, we embed each motif occurrence in a sequence with varying flanking sequence lengths. The flanking sequences were unrelated to the motif families. Given a set of such sequences, the goal was for the algorithms to correctly recover the family secondary structure motifs. We also consider an alternate version of the benchmark where we introduced entirely random unrelated sequences into the sequence set. Together, the two benchmarks approximate the setting where users are interested in identifying local RNA secondary structure motifs embedded in longer RNAs (such as mRNAs), in spite of the presence of a few entirely spurious, unrelated RNAs among the input sequences.

Of the stochastic algorithms discussed, MEMERIS can only infer linear motifs in single stranded regions and cannot discover secondary structures explicitly. Hence we consider CMfinder and RNApromo for benchmarking in this category. Yao et al. [32] demonstrate that CMfinder significantly outperforms comRNA on a variety of families from Rfam database. Hence, among the stem methods, we only consider RNAmine for comparisons. Among the alignment based methods, FOLDALIGN and its variants cannot tackle sufficiently general secondary structures on more than two input sequences. The LocARNA in principle can compute structure-based multiple local alignments, but the current implementation of LocARNA doesn’t support the output of such multiple local alignments. Hence among secondary structure independent alignment algorithms, we look at SCARNA_LM only. The secondary structure dependent alignment algorithms we have discussed so far need information about the complete secondary structure of the input sequences. This information on the one hand can be inaccurate if computational methods are used and on the other hand can be quite expensive with experimental methods. Also, given that we use extraneous flanking regions around the motif occurrences in input sequences, folding such sequences may not make realistic sense in some cases. Hence we do not consider any of these methods. Among the miscellaneous methods, the tree mining approach of [55] concentrates on the design of a general frequent tree algorithm and uses RNA based data as a minor application. This approach would be reasonable if one knows the true secondary structures of the input sequences and a method to score the output patterns. The genetic programming approach GPRM needs information about the presence or absence of the motif in every input sequence which can be an unreasonable requirement. Shahar et al. [59] demonstrate the superior performance of the GeRNAMo over the ad hoc RNAProfile. GeRNAMo would be the algorithm to compare with but there is no online implementation of GeRNAMo available. For the above mentioned reasons, we do not consider any of the miscellaneous methods for benchmarking. To summarize, we consider four methods, namely CMfinder, RNAPromo, ScarnaLM and RNAmine for benchmarking and performance comparisons.

To study the performance, we use a single measure based on the standard sensitivity and positive predictive value (PPV) measures of structure prediction. Given a reference (true) structure and a predicted structure, sensitivity is defined as the fraction of the base pairs in the reference structure which are predicted correctly. PPV refers to the fraction of the predicted base pairs which are also present in the true structure. To be a bit more precise, we introduce true positives (TP), false positives (FP) and false negatives (FN) in this context. TP refers to those base pairs in the reference structure which are also predicted correctly. FP refers to those base pairs which are predicted but absent in the reference structure. FN represents the number of base pairs in the reference structure that are not predicted. With these quantities, sensitivity can be defined as $\frac {TP}{TP + FN}$ and PPV as $\frac {TP}{TP + FP}$. The best case scenario happens when both sensitivity and PPV are close to 1. Given this, one natural measure is to consider the geometric mean of sensitivity and PPV, also referred to as prediction accuracy. In such a case, prediction accuracy is also close to 1. Unlike structure prediction (from a single sequence), in our context we have multiple sequences. Hence while calculating sensitivity and PPV for the sequence set, we use the total number of true positives, false positives and false negatives calculated across all the sequences.

In this section, we basically study the effect of noise on performance of the different algorithms. In the first experiment, we study the effect of increasing the length of extraneous flanking segments on either side of the motif locations. The alignments in Rfam are on genome segments which exactly correspond to the motif locations. To obtain sequences with extraneous flanking regions, we had to separately download genome segments (around the motifs in Rfam) from GenBank with appropriate additional flanking. We progressively vary the length of the extraneous flanking region (*L*_*fℓ*_), which refers to the combined length of the left and right flanking segments around the motif location. The length of the left flanking segment is randomly chosen between 0 and *L*_*fℓ*_ for each sequence.

**Fig. 15:**
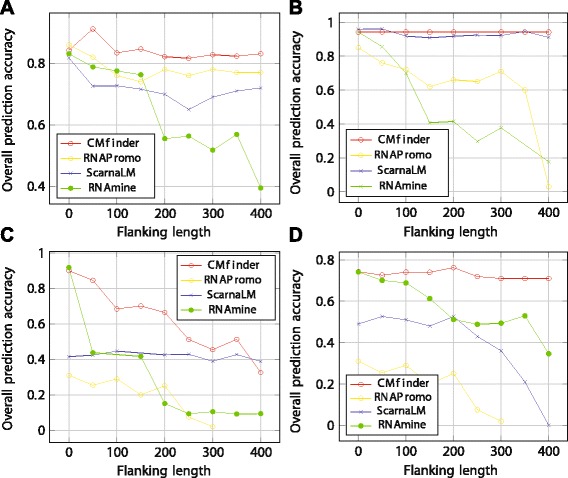
Performance as function of flanking length. Four Rfam motifs were embedded in sequences with increasing flanking length (x-axis, nucleotides). The y-axis gives the methods’ performance at correctly identifying the embedded motif. Rfam motifs: (**a**) let-7, (**b**) Purine, (**c**) RFN, (**d**) glmS

Figure 15 gives the performance comparison of the four algorithms. Each subfigure represents the performance comparison on a particular pattern. As can be seen from all the figures, CMfinder has better performance uniformly over the other methods. RNAPromo and ScarnaLM perform reasonably well on the shorter patterns let-7 and Purine but exhibit poor performance on the longer patterns RFN and glmS. RNAmine performs well with little or no flanking region, but surprisingly exhibits a poor performance with increase in the flanking length.

**Fig. 16:**
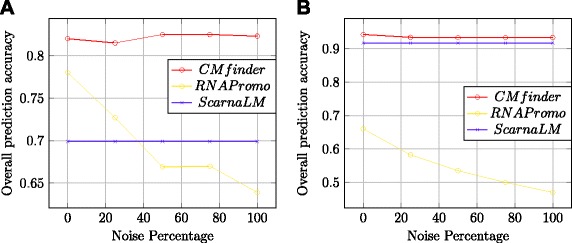
Performance as function of noise level. Two Rfam motifs were embedded in 200 nucleotide long flanking sequences and increasing percentages of unrelated sequences were added to the collection (x-axis). The y-axis gives the methods’ performance at correctly identifying the embedded motif. Rfam motifs: (**a**) let-7, (**b**) Purine

In the second experiment, in addition to flanking we introduce entire separate sequences with no related motifs. We study the effect of adding entirely unrelated noisy sequences to the set of related sequences from a particular family. Each such noise sequence was a segment of DNA from GenBank containing a different (randomly chosen) motif’s occurrence. We study the effect on prediction accuracy of increasing the number of such unrelated noisy sequences. The flanking lengths were fixed at about 200 among the sequences containing the motif. Figure 16 displays the performance variation with increase in noise sequences (expressed as a percentage of motif-containing sequences). We didn’t include RNAmine in this experiment as its performance was pretty poor in the previous experiment with increase in length of flanking regions. The experiment indicates that CMfinder and ScarnaLM in particular are very robust to addition of noise sequences.

## Discussions and conclusions

In this paper, we have focused on the problem of discovering secondary structure based local patterns from a set of related RNA sequences. We reviewed the various computational approaches proposed in literature to tackle this generic problem. The landscape of methods was quite diverse with approaches based on stochastic learning, pattern mining, genetic programming, DP and so on. In addition to the diversity in computation, one can also see substantial variety in the manner in which motifs are defined. MEMERIS looks for stochastic sequence based motifs in preferably single stranded regions. CMfinder and RNAPromo discover motifs (SCFG-based stochastic models) which are both sequence and secondary structure based. The stem based methods look at motifs as a certain (one of three) combination of stems with a purely secondary structure based focus. The genetic programming-based methods we discussed discover motifs as a sequence of segments which are either single stranded or complementary, with a bound (upper and lower) constraint on their lengths. These motifs are also solely secondary structure based. Motifs as per the tree mining approach of [55] would be a tree composed of nodes corresponding to basic secondary structure motifs like stems and the various types of loops. Most of the alignment based methods we discussed discover motifs based on both sequence and structure similarity. Most methods we discussed here searched for motifs whose occurrences were contiguous subsequences of the input sequences. We discussed two pairwise alignment methods (based on both sequence and structure as input), which discover sequence structure based motifs whose occurrences may not translate to contiguous subsequences in the input sequences.

Predicting RNA secondary structure from RNA primary sequence is a classic and important problem in RNA analyses, as this has been the only viable approach for high throughput analyses and inference of RNA structure. Recently, several groups have developed experimental techniques that allow high throughput analyses of RNA structures, both *in vitro* and *in vivo* (see [62] for a review). Although the exact details of these techniques vary, the methods have the following three main steps in common: (i) treat RNA such that either base-paired or single stranded nucleotides are tagged, (ii) use high-throughput sequencing of treated RNA to identify tagged nucleotides, and (iii) process sequencing data to identify regions in the input RNA that are enriched for base-paired or single stranded nucleotides. In the first step, current methods either use chemical or enzymatic treatment for tagging. The chemical-based methods give single-nucleotide data on structure, but currently only allow tagging of single-stranded regions of RNA. In contrast, the enzymatic methods use ribonucleases that either cleave double- or single-stranded regions of RNA and thereby allow tagging of both structure types. But as the enzymes are bulky and can be too large to interact with and cleave all the double- or single-stranded regions in RNA, enzymatic methods give lower-resolution data than do chemical-based methods. Currently, only chemical-based methods are available for *in vivo* analyses.

Irrespective of the experimental details, the output from these methods are essentially RNA stability profiles that for each RNA present in the input sample, identify regions that are more or less likely to contain base pairs. Notably, although these stability profiles identify nucleotides that participate in base-pairing, the profiles do not directly reveal the identity of these base pairs. Instead, RNA structure prediction programs use the stability profiles as additional constraints to identify the structure that is most likely to correspond to the observed stability profile. The simplest approach is to interpret the stability profile as hard constraints in a standard MFE structure prediction algorithm; that is, nucleotides that according to the profile are single- and double-stranded are forced to be single- and double-stranded, respectively, in the structure prediction algorithm. This is the approach taken in for example the SAVoR web tool [63]. The better approach, however, is to use the stability profile as soft constraints; for example, as an additional pseudo-free energy change term in the MFE structure prediction algorithm [64]. Using this approach, Deigan et al. achieved much better predictions than when using stability profiles as hard constraints or when using MFE structure prediction alone [64]. We expect that incorporating such experimentally determined stability profiles as input to RNA motif discovery algorithms will lead to similar improvements. The most straightforward approach would be to do motif discovery on sets of stability profiles – for example, by running MEME on discretized stability profiles. Similarly, stability-profiles can easily replace the base-pairing probabilities from Boltzmann ensembles in the MEMERIS algorithm. However, extending, for example, CMfinder to include stability profiles as an additional pseudo-free energy change term, similar to [64], would likely give the best results.

Among the various kinds of motifs considered in literature, one would ideally want patterns to capture a combination of sequence and structure similarity which in a sense is captured by stochastic methods like CMfinder and RNAPromo and most of the alignment methods. However these methods fail to capture structure similarity involving pseudoknots, which are otherwise capturable by the stem-based methods like comRNA and RNAmine, and also by GPRM, the genetic programming-based method. But these latter methods do not capture any sequence based similarity. All the other methods we discussed can only discover nonpseudo-knotted motifs. Accordingly, there are currently no methods which can unearth pseudoknot based local patterns involving both sequence and structure similarity. This is a very important direction for future research that needs to be pursued. In the previous section, RNAmine showed reasonable performance with only little or no flanking regions around the motif occurrences. It was also demonstrated in [38] that RNAmine ran much faster than CMfinder, a state-of-art method with the best prediction accuracies for this problem. Given RNAmine’s ability to handle pseudoknots and superior runtimes, improving upon RNAmine to handle larger flanking regions would be a noteable enhancement. All the methods which can handle multiple sequences without any structure input can only capture motifs whose occurrences are necessarily contiguous subsequences. This requirement can be a restriction [54] and designing methods to tackle this is another useful direction for future research.

## Reviewers’ comments

### Reviewer 1, first report: Dr. Sebastian Maurer-Stroh, Bioinformatics Institute, A*STAR, Singapore

This manuscript aims to review and partially test computational tools for RNA motif discovery. Overall language editing may make the text more accessible although it is possible to follow also as it is.

A dedicated section gives an introduction to RNA secondary structures including typical graphical representations. However, not covered here are for example the mountain plot as well as the dot plot. Especially the latter allows to visualize ensemble base pair probabilities which are also relevant in this context as some of the described methods utilize partition functions in the folding algorithms. Please consider adding/discussing this, including a simple description of what RNA secondary structure ensembles are.

Authors’ response: *We fully agree with the reviewer on this point. We have now added material on dot plot and RNA secondary structure ensembles towards the end of the RNA Secondary Structure section.*

The next section tries to roughly classify existing computational approaches and then introduce the underlying ideas and algorithms. Although there will always be more methods that one could consider and the full intricacies of a method cannot be summarized in a few sentences, the authors nevertheless provide a relatively reasonable overview without too much complicated details which is rare to find in style of a side by side comparison of multiple methods. I consider this section useful.

Authors’ response: *We thank the reviewer for the positive comments on this section.*

Finally, a short benchmark is presented over 4 selected RNA motifs from Rfam. Testing influence of the natural or artificial flanking regions of the motifs with varying length is a good idea. However, I would like to make some remarks here. A common scenario of trying to discover RNA motifs will have as input an unaligned set of candidate sequences of varying length (or same length but different positions of motifs along the sequence). Providing a test motif with symmetric flanking regions of same length on either side, makes it easy to align the motif in the middle (more challenging would be randomly differing flanking lengths or motifs located in different regions of the input sequences). Another typical scenario would be an alignment of closely related homologous sequences which will necessarily show sequence and structural conservation over a longer region than just the typical single RNA motif length. If the flanking regions of the test motif are not directly related to each other as in this benchmark it will be much easier to find the motif. As analogy, the benchmark setup can be compared to finding a tree on a plain field while the homologous alignment scenario would be more like finding the right tree in a forest. It is not clear, if both more challenging scenarios can be emulated for this review but it would be good if some thought could be given to it.

Authors’ response: *The two scenarios pointed out by the reviewer make a lot of sense. However, the first scenario pointed out above, where flanking regions need not be symmetric on either side of the motif, is precisely what has been incorporated in the paper. Paragraph number 5 in the Performance Comparison section conveys this point. So, even though the sum of the flanking lengths (denoted asL*_*fℓ*_*) on either side are the same in all sequences, the motif position in each of the input sequences is randomly chosen and hence different in each input sequence. Regarding the second scenario, we have not addressed this directly. However, our flanking regions are not artificial (or randomly generated), but the true genomic flanking regions around the motif location. It is intuitive to expect that having natural flanking regions can be more challenging than having totally random flanking regions. Given this, we feel the benchmark setup in this paper is sufficiently non-trivial.*

Minor comments/typos:

- p.1 Abstract: “This class of methods essentially learn” should be “This class of methods essentially learns”
- p.2 first line: “from from”
- p.2 last paragraph: the point of a “riboswitch” is that it changes/switches the structure under specific conditions (binding, temperature,...) which is not at all clear in this sentence.
- p.7 second paragraph: “each of this class” should be “each of this classes”
- p.12: the CMfinder reference appears late instead of at the beginning of the respective method description
- p. 27 third paragraph: “GeneBank” should be “Genbank”

Authors’ response: *We have made the necessary corrections and clarifications.*

### Reviewer 1, second report: Dr. Sebastian Maurer-Stroh, Bioinformatics Institute, A*STAR, Singapore

I am ok with the replies to my comments.

### Reviewer 2, first report: Dr. Erez Levanon, Faculty of Life Sciences, Bar-Ilan University, Ramat-Gan, Israel

The review “RNA motif discovery: a computational overview” by Achar and Satrom deals with the various approached to detect RNA motif. The review is a thorough, detailed comparison of the current computational approaches in this field. For people like me, that lack the relevant background in algorithms of RNA structure predication, it can serve as a reference and a good summary. Especially useful is the benchmark part at the end of the review.

Authors’ response: *We thank the reviewer for the kind words.*

The review is almost entirely algorithmic, and I would suggest, in order to make it more relevant for biologist, to add some biological insight, for example: How the different approaches deal with various types of RNA motifs? Moreover, the field of RNA structure predication is in the process of revolution due to the developing of new sequencing-based methods. It is important to discuss the impact of the new technologies on the landscape of RNA motif discovery.

Authors’ response: *We have explained in relative detail in the first paragraph of the*Discussions and conclusions*section about what kind of motifs are extracted by the various approaches discussed in this paper. Also, the third paragraph of the Performance Comparison section touches upon this point. We have added a short discussion in the*Discussions and conclusions*section on the possible impact of new sequencing technologies on RNA motif discovery.*

In addition, there are several minor issues that should be fixed in the next version. For example:

- The last paragraph of section 2 is not clear- who is “they”? (page 7)
- Page 10 “The paper demonstrate” not clear which paper
- Page 17: first sentence of section 3.3.1 is not clear

Authors’ response: *We have made the necessary corrections.*

### Reviewer 2, second report: Dr. Erez Levanon, Faculty of Life Sciences, Bar-Ilan University, Ramat-Gan, Israel

The authors address the issues raised regarding the previous version of the manuscript (although I would consider elaborating more about sequencing based methods due to their impact on the field).

Authors’ response: *As suggested, we have further elaborated on the sequencing-based methods.*

### Reviewer 3, first report: Dr. Weixiong Zhang, Department of Computer Science and Engineering, Washington University, St. Louis, Missouri, USA

This reviewer provided no comments for publication.

## Abbreviations

ChIP: Chromatin immunoprecipitation
CM: ovariance models
CRF: Conditional random field
DP: Dynamic programming
EM: Expectation maximization
FN: False negatives
FP: False positives
HMM: Hidden Markov model
i.i.d: Independent and identically distributed
MEME: Multiple EM for motif elicitation
MEMERIS: MEME in RNAs including secondary structures
MFE: Minimum free energy
mRNA: Messenger RNA
ncRNA: Non-coding RNA
OOPS: One motif occurrence per sequence
PPV: Positive predictive value
PWM: Position weight matrix
rRNA: Ribosomal RNA
SCFG: Stochastic context-free grammars
SECIS: Selenocysteine insertion sequence
TP: True positives
tRNA: Transfer RNA
UTR: Untranslated region
ZOOPS: Zero or one motif occurrence per sequence
